# Digital Image Correlation Compatible Mechanoluminescent Skin for Structural Health Monitoring

**DOI:** 10.1002/advs.202105889

**Published:** 2022-02-13

**Authors:** Ho Geun Shin, Suman Timilsina, Kee‐Sun Sohn, Ji Sik Kim

**Affiliations:** ^1^ Department of Advanced Science and Technology Convergence Kyungpook National University 2559, Gyeongsang‐daero, Sangju‐si Gyeongsangbuk‐do 37224 Republic of Korea; ^2^ School of Nano & Advanced Materials Engineering Kyungpook National University 2559, Gyeongsang‐daero, Sangju‐si Gyeongsangbuk‐do 37224 Republic of Korea; ^3^ Nanotechnology & Advanced Materials Engineering Sejong University 209 Neungdong‐ro, Gwangjin‐gu Seoul 143‐747 South Korea

**Keywords:** crack‐tip HRR field, mechanoluminescent skin, mechanoluminescent (ML) quantification, structural health monitoring, trap‐controlled mechanism

## Abstract

Monitoring structural health using mechanoluminescent (ML) effects is widely considered as a potential full‐field and direct visualizing optical method with high spatial and temporal resolution and simple setup in a noncontact manner. The challenges and uncertainties in the mapping of ML field to effective strain field, however, tend to limit significant commercial ML applications for structural health monitoring systems. Here, however, quantification problems are resolved using the digital image correlation (DIC) method. Specifically, an image containing mechanically induced photon information is processed using a DIC algorithm to measure the strain field components, which enables the establishment of a calibration curve when the ML field is mapped onto the effective strain field using pixel level information. The results show a linear relationship between effective strain and ML intensity despite the plastic flow in ML skin. Furthermore, the calibration curve allows for easy conversion of ML field to effective‐strain field at the crack‐tip plastic zone of the alloy structure, retaining its spatial resolution. The compatibility of ML skin with the DIC algorithm not only enables the quantification of the ML effects of several organic/inorganic ML materials, but may also be useful in elucidating the fundamentals of the trap‐controlled mechanism.

## Graphical abstract

The compatibility of mechanoluminescent (ML) skin with the digital image correlation under UV exposure is leveraged to quantify the ML effects in terms of effective strain. The calibration can be executed using the same photographic image. Interestingly, a linear correlation exists despite the plastic flow in ML skin. The quantification extends the ML application from visualizing to quantitative measurement.

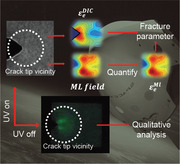

## 1 Introduction

The structural components used in automobiles, aeronautics, astronautics, and marine industries are subjected to severe mechanical environments during their service life. To satisfy the requirements associated with service in extreme loading environments, high‐fatigue strength metallic alloys with a high strength‐to‐weight ratio are used. However, the presence of defects such as voids during manufacturing, joints of welded components, and edges of complex geometrical structural components tends to amplify the effective strain in their vicinity, which affects the structural load bearing capacity owing to the initiation and advancement of cracks from the strain‐amplified areas. In general, it is necessary to identify and monitor the effective strain concentration zones before a critically strained state is reached. The inability to inspect the location of the effective strain concentration zones before they reach a critically strained state could lead to catastrophic failure of the structure, which could result in the loss of human life. Therefore, full‐field, noncontact, and in situ strain measurement technology is extremely important to monitor the integrity of structures and components.

Conventionally, force‐ and displacement‐driven voltage signals are measured using piezoresistive, piezoelectric, and piezocapacitive sensing devices to measure the average values of local strain components in different directions.^[^ ^1^ ^]^ However, full‐field inspection cannot be realized using voltage signals because printing of integrated circuits for geometrically large structures is highly challenging and can be prohibitively expensive.^[^ ^2^ ^]^ In recent decades, deformation measurement based on optical methods has become increasingly popular because of the ease of recording photons either reflected by or emitted from the structure using an imaging system, which can enable noncontact, nondestructive, and full‐field examination. Optical methods based on the use of moiré patterns, interferometry, and holography have been used to conduct surface deformation measurements.^[^ ^3^ ^]^ However, the application of these methods to structural health monitoring is limited owing to the complicated processes involved in sample preparation and data acquisition, the need for sophisticated equipment, and inadequate local or global spatial resolution.^[^ ^4^ ^]^ In recent years, infrared thermography has been used extensively in industrial applications as a full‐field and noncontact optical method to visualize the evolution of the effective stress and strain fields in terms of the temperature distribution. However, the need for a constant background temperature limits the use of this approach in controlled environments. Digital image correlation (DIC) is another optical method that has been used exclusively in industrial applications to measure displacement field components and strain field components.^[^ ^5^ ^]^ However, DIC is an indirect visualization technique that relies primarily on a complex algorithm that requires a high level of computational performance.

To overcome the above limitations, researchers have focused on developing a novel optical method using mechanoluminescence (ML), which can enable global and local inspection with high spatial and temporal resolution in a noncontact manner at low cost to identify and monitor the effective strain concentration zones.^[^ ^6^, ^7^, ^8^, ^9^ ^]^ In ML technology, the surface distribution of the effective strain on a structure and its components are visualized based on mechanically induced photons from the application of an artificial skin composed of nano‐ and micro‐ML particles and a suitable polymer matrix. Theoretically, individual ML nano‐ and microparticles function as strain sensing probes. Despite historical documents revealing that the ML phenomenon was discovered four centuries ago, only in the past two decades has the pioneering work of Xu and co‐workers ignited ML research.^[^ ^8^, ^10^ ^]^ Their seminal work focused on the possibility of developing a low cost stress sensor using the ML of SrAl_2_O_4_ (SAO):Eu and ZnS:Mn. Different classes of ML materials have been discovered and applied in several domains since then, such as displays, mapping of personalized handwriting, pressure memory, ultrasound visualization, and heartbeat monitoring.^[^ ^11^, ^12^, ^13^, ^14^, ^15^ ^]^ In the field of fracture mechanics, the potential of ML technology in crack‐tip position identification, crack‐tip effective stress, and strain field visualization in elastic and plastic fields, crack path prediction based on the crack‐tip effective stress field, and hidden crack identification in pressurized hydrogen fuel cells has been demonstrated.^[^ ^16^, ^17^, ^18^, ^19^, ^20^ ^]^ However, the problem of calibrating the ML intensity and strain must be addressed before ML technology can be considered a reliable and robust method for structural health monitoring of megastructures and their components. The first challenge in the calibration is to segregate the ML from the inherent decay of the phosphorescence (PL) after UV preirradiation.^[^ ^6^, ^7^, ^8^, ^9^ ^]^ The second challenge is to measure the effective strain at various points on the surface of a uniaxial tension specimen without affecting the emissions from the ML skin. In the conventional approach, the loading direction strain component is measured using a strain gauge or an extensometer to correlate it with the ML intensity.^[^ ^21^, ^22^, ^23^ ^]^ Consequently, the impact of other strain components is ignored in the correlation. Moreover, measurement of strain and ML emission from the same position is not possible using the conventional approach. To address the first challenge, Sohn et al. demonstrated that continuous UV excitation can be used to manage the effect of decaying PL by maintaining a constant photoluminescence background.^[^ ^24^ ^]^

**Figure 1:**
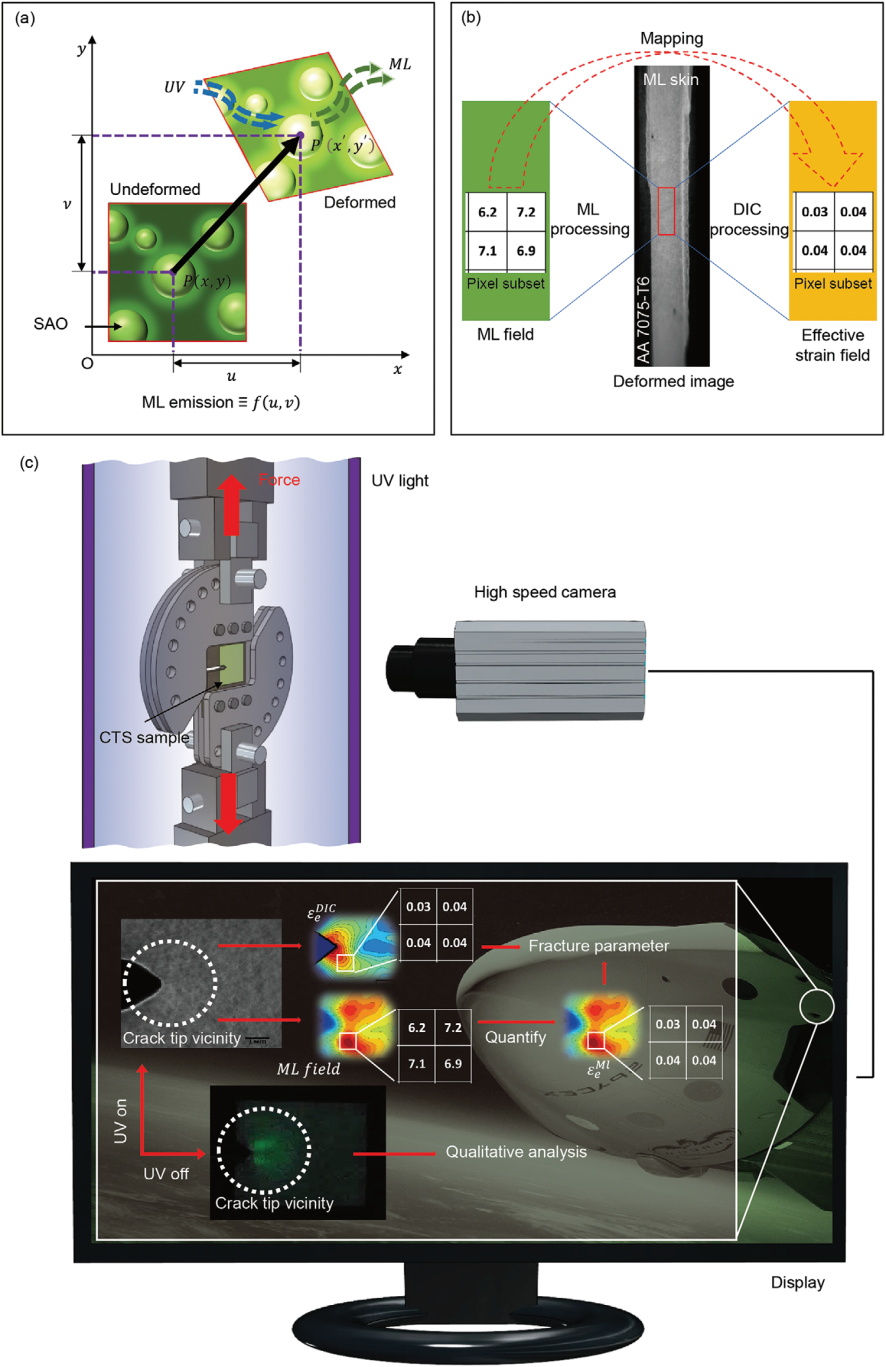
Correlative structural health monitoring using ML and DIC methods. a) Schematic diagram of ML skin functioning when the DIC and ML methods were used to measure the deformation. b) Schematic illustration of the ML field for effective strain mapping using pixel level information of the same image obtained from uniaxial tension test. c) Schematic diagram of a CTS specimen inserted at the loading stage with the video recorded during loading under continuous UV exposure. The recorded grayscale images are processed separately using ML and DIC methods to visualize the crack‐tip vicinity ML field and effective strain field, respectively. Furthermore, the quantified ML field ($\varepsilon_{e}^{\mathrm{ML}}$) and DIC‐measured effective strain field ($\varepsilon_{e}^{\mathrm{DIC}}$) can be used to measure the fracture parameters. Importantly, the conventional method of visualizing the crack‐tip deformation field in terms of the ML field after UV preirradiation is also feasible using the ML skin of the present study.

In this study, we show that the second challenge in quantification of ML could be resolved using the DIC method. We found that the ML skin, which is composed of SAO:Eu,Dy ML microparticles and plasticized epoxy matrix, has the necessary variation in local intensity pattern under UV exposure available for DIC algorithm to measure displacement fields and strain field components (**Figure** **1**a). Therefore, the compatibility of ML skin with the DIC algorithm can be leveraged to quantify the ML effects in terms of DIC‐measured effective strain. In this study, we applied ML skin on the aluminum alloy (AA7075‐T6, which is commonly used in the aerospace and automobile industries) tension specimen. By doing so, the ML phenomenon as well as the measurement of the effective strain field could be monitored using the same photograph, and the calibration curve showing the correlation between ML and effective strain throughout the flow curve of the specimen could be obtained (Figure 1b). The use of plasticizer enhanced the stretchability of ML skin to comply with the large deformation (12%) of the specimen. Impressively, linear correlation exists despite the plastic flow in the specimen and ML skin. The calibration curve from a simple deformation field of uniaxial tension testing can be used to convert the complex singularity‐dominated crack‐tip ML intensity field into an effective strain field. In this regard, we visualize (for the first time) the evolution of the plastic zone in terms of ML at the crack‐tip vicinity of AA7075‐T6 and subsequently quantify it in terms of effective strain field. This transformation of the ML field into an effective strain field further allows the determination of structural integrity parameters (fracture parameters) such as the *J*‐integral using Hutchinson–Rice–Rosenberg (HRR) near‐tip singularity solution (Figure 1c). Moreover, the possibility of extending the DIC method to directly measure the singularity‐dominated effective strain field and determine the fracture parameters can be crucial in validating measurements using the ML method (Figure 1c). Furthermore, the ML skin used in this research can also function within the framework of conventional ML technology, where UV is turned off following preirradiation (Figure 1c). The high contrast in situ ML images obtained during UV cessation help identify the strain concentration zones on a structure prior to quantitative analysis under UV irradiation. This work will open the door for quantifying ML effects of several organic/inorganic ML materials that are considered for structural health monitoring systems, motion biometric, artificial photonic skin, displays, and other ML applications, which inevitably extends the application of ML from visualization to quantitative measurement.^[^ ^8^, ^9^, ^19^ ^]^

## 2 Experimental Section

### 2.1 Materials

Green photon‐emitting mechanoluminescent SAO:Eu,Dy with excitation and emission peaks of 360 and 530 nm, respectively, was purchased from Nemoto & Co., Japan. Epoxy‐1050, hardener‐1056S, and plasticizer‐1600/‐1606 were purchased from Resoltech, USA. Furthermore, a 3 mm aluminum alloy AA7075‐T6 sheet was purchased in a general market in South Korea. AA7075‐T6 was considered in this study because it was frequently used in aerospace and automobile structural components owing to its high strength‐to‐density ratio and high ductility characteristics.

### 2.2 Fabrication of the Mechanoluminescent Composite

The ML skin was fabricated by mixing three different components: SAO, epoxy resin, and plasticizer. The SAO constituted 30 wt% of the total weight of the epoxy and plasticizer. The epoxy and plasticizer were mixed at a ratio of 6:4 by weight. A plasticizer was added to enhance the ductility of the skin, and the ratio was selected to match the failure strain of the AA7075‐T6 substrate. Mixing was performed in a planetary mixer for 5 min; thereafter, the resultant homogenous mixture was degassed for 10 min, as illustrated in Figure S1 (Supporting Information).

### 2.3 Coating of the Mechanoluminescent Composite on the Substrate

Before applying the well‐mixed composite onto one of the surfaces of the compact tension shear (CTS) and tension specimens, the specimen surface was cleaned with H_2_SO_4_ followed by acetone. Surface treatment was important to improve adhesion of the composite to the surface. The region for the ML skin was separated using Scotch tape with a thickness of 80 µm, and the composite was spread over the region of interest using the doctor blade technique. The samples were then transferred to a vacuum oven and left to dry for 12 h at 60 °C, which resulted in a well‐adhered ML skin on the surface of the AA7075 T6 with a thickness of ≈100 µm.

### 2.4 Tension Test

**Figure 2:**
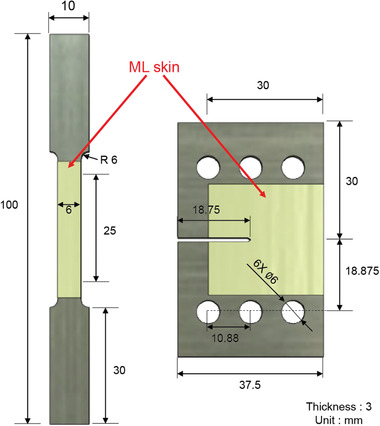
Dimensions of the uniaxial tension and CTS specimens. The thickness of the ML skin was ≈80 µm.

For the uniaxial tension test, a tension sample with the dimensions illustrated in **Figure** **2** was transferred to an Instron machine (model 5567) equipped with a specially designed tension‐type loading stage. The specimen was then irradiated using a constant UV‐360 nm source (1500 lux, INNO‐CURE 500, South Korea) and the sample was loaded at a crosshead speed of 0.05 mm s^−1^ with continuous UV exposure. The region of interest at the gauge section was photographed using a high‐speed imaging system (FASTCAM SA‐X2, Japan) at a speed of 125 fps throughout the loading. A multichannel data link was used to ensure perfect synchronization between the load data from the load cell and recorded images.

### 2.5 Fracture Test

For the fracture test, a CTS sample with a thickness of 3 mm, initial crack length of 18.75 mm, and width of 37.5 mm was transferred to an Instron machine (model 5567) equipped with a specially designed CTS‐type loading stage, as shown in Figure 1b. The detailed dimensions of the CTS sample are illustrated in Figure 2. The load was applied such that mode I fracture was achieved. The experimental conditions, such as UV irradiation, frame speed, and crosshead speed, were the same as those of the uniaxial tension test.

### 2.6 Statistical Analysis

**Figure 3:**
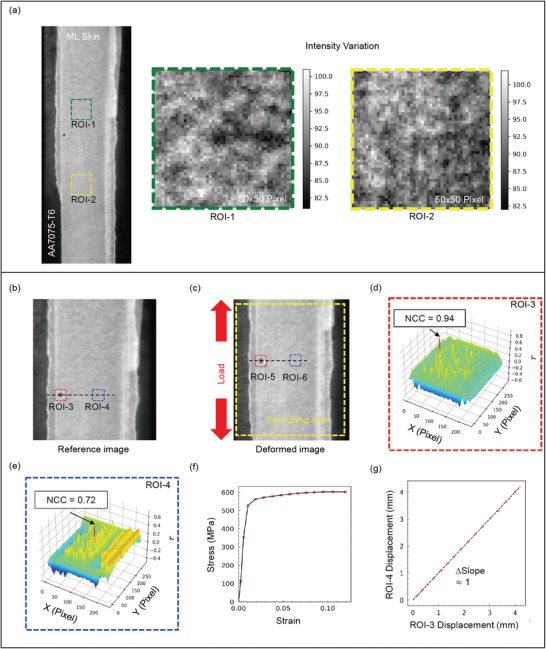
Compatibility of ML skin with the DIC method for deformation measurement. a) Variations in the local intensity pattern of ML skin under UV exposure are highlighted by selecting two differently located pixel windows (ROI‐1 and ROI‐2) in the gauge section of the tension specimen. b) Reference image at zero external load where ROI‐3 and ROI‐4 were selected as the reference subsets. c) Deformed image where ROI‐5 and ROI‐6 are the deformed subsets of the reference subsets ROI‐3 and ROI‐4, respectively. d) 3D plot showing the NCC distribution for ROI‐3. e) 3D plot showing the NCC distribution for ROI‐4. f) True stress–strain flow curve of AA7075‐T6. g) Comparison of the displacement measurement for the reference subsets ROI‐3 and ROI‐4 by considering several images representing the different deformation states as marked in the true stress–strain curve.

**Figure 4:**
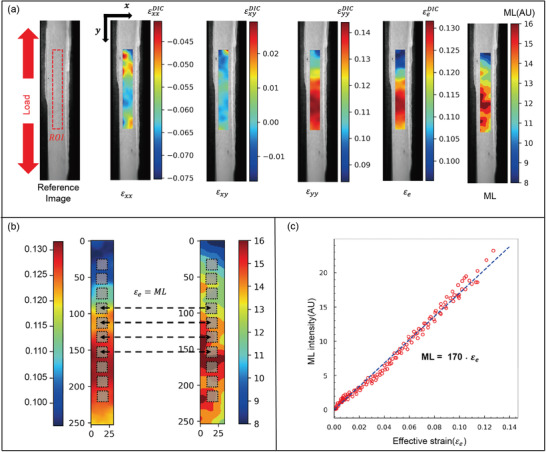
Calibration of ML intensity and effective strain. a) The leftmost image shows the reference image with the defined region of interest (ROI) of 250 × 28 pixels at zero external load; the rest of the images, respectively, represent the DIC‐measured strain components (*ε*_*xx*_, *ε*_*xy*_, and *ε*_*yy*_), the effective strain (*ε*_e_) calculated from the strain components, and the ML intensity field. b) The pixel subsets of *ε*_e_ and the ML field where the respective field was averaged out before one‐to‐one mapping between the ML intensity and *ε*_e_. c) The calibration curve obtained from the linear regression fit of the scattered plot between ML and *ε*_e_.

The determination of normalized cross‐correlation coefficient in **Figure** **3** between the reference and the deformed images was conducted in MATLAB. The linear regression model was used in fitting the data in **Figure** **4**c using Python. Absolute error was considered to compare the *J*‐integral obtained from DIC and ML methods considering finite element method (FEM) as the standard in **Table** **1**; Python was used for calculating the absolute error.

**Table 1:** Comparison of the *J*‐integrals measured using DIC, ML, and FEM methods. The table also presents the absolute error (AE) considering the FEM *J*‐integral as the reference value

*J*‐integral [kJ m^−2^]		*P*_1_	*P*_2_	*P*_3_	*P*_4_
*J*_FEM_		4.28	20.69	32.11	49.11
*J*_DIC_		4.89	20.49	26.17	42.19
*J*_ML_		5.73	20.01	32.87	49.06
AE^a)^	ǀ*J*_DIC_ − *J*_FEM_ǀ	0.61	0.2	5.94	6.92
	ǀ*J*_ML_ − *J*_FEM_ ǀ	1.45	0.68	0.76	0.05

^a)^ AE: absolute error.

## 3 Results and Discussion

### 3.1 Compatibility of ML Skin for Use with DIC Technology

An artificial speckle pattern or the natural texture on the surface of a structure is essential for the application of DIC as a carrier of deformation information, which enables tracking of the motion of the pixels of concern from the reference image of the deformed substrate.^[^ ^25^ ^]^ Conventionally, a speckle pattern is created by spraying black or white paint depending on the color of the substrate, which results in variations in the local intensity. The variations in local intensity are an important trait that the DIC algorithm processes to find the transformed position after deformation. Fortunately, ML skin exhibits variations in local intensity under UV exposure due to the photoluminescence of SAO particles, which absorb UV light and emit photons at a peak wavelength of 530 nm. The inhomogeneous distribution of SAO particles and variations in the particle size distribution (Figure S2, Supporting Information) creates an inherently random intensity pattern at the local level in the ML skin. These variations are illustrated in Figure 3a considering two regions of interest (ROI‐1 and ROI‐2) with a size of 50 × 50 pixels. The gray color maps clearly illustrate the random intensity patterns in the ML skin. It should be noted that no external load was applied in Figure 3a, so the local intensity variation is solely from PL. The microlevel inhomogeneous distribution of the intensity of the ML skin can be exploited for deformation measurement using the DIC principle, in addition to other important deformation information encrypted in the mechanical‐stimuli‐triggered photon emission.

When using the DIC technique, a reference subset containing sufficient local intensity variations centered at the pixel of interest is selected and identified in the deformed image by calculating the normalized cross‐correlation coefficient (NCC). The maximum NCC indicates the maximum similarities in the intensity pattern, which can help identify the position of the pixel of interest in the deformed image. In the present study, we attached ML skin to a section of AA7075‐T6 aluminum alloy and conducted a uniaxial tension test to determine the NCC. A reference image corresponding to zero external load and a deformed image were selected, as shown in Figure 3b,c, respectively. Subsequently, a size 50 × 50 reference subset, ROI‐4, was selected in the reference image, and its position was identified in the searched area of the deformed image. Moreover, an additional 50 × 50 reference subset, ROI‐3, was selected adjacent to ROI‐4, which included an artificially created circular hole. A circular hole was created on the ML skin to increase the contrast in the ML skin, enabling visual tracking of the position and verifying the position established using NCC information.

The NCC was calculated based on the algorithm proposed in ref. [26] and was implemented using MATLAB. In the range of the obtained values [0, 1], “1” represents the maximum similarity and “0” represents no similarity. Normalization compensates for any errors associated with the deformation‐induced intensity increase of the ML skin.^[^ ^26^ ^]^ The NCC calculated for ROI‐3 is ≈0.94, whereas that for ROI‐4 is ≈0.72, as indicated in Figure 3d,e, respectively. As expected, the higher contrast in ROI‐3 resulted in a higher NCC value; however, the tracked positions of ROI‐3 and ROI‐4 were determined based on NCC information, as illustrated in the deformed image in Figure 3c as ROI‐5 and ROI‐6, respectively. It can be observed that the center points of ROI‐5 and ROI‐6 are almost collinear as they are in the reference image. This clearly suggests that the ML skin exhibits sufficient local intensity variations even without the aid of an artificial contrast agent, which is compatible with the DIC technique when measuring displacement vectors.

The deformed image shown in Figure 3c corresponds to an external load of ≈100 MPa, which is well within the elastic limits of AA7075‐T6, as illustrated in the stress–strain curve in Figure 3f. Because the present study focused on plastic deformation, the compatibility of ML skin with the DIC technique to the point of failure for AA7075‐T6 is significant. Therefore, loading direction displacements were calculated for both subsets at different stages of loading based on the procedure described above, as indicated in the true stress–strain flow curve. Figure 3g shows the measured displacements for both subsets, and the results are identical despite the extremely high NCC of ROI‐3. These findings demonstrate that the ML skin can be used to measure even larger deformations (12% strain) in a reliable and robust manner based on the DIC algorithm. Furthermore, ML during loading does not seem to interfere with the DIC algorithm's ability to determine the NCC based on variation in the local PL intensity. This may be due to the very small ML intensity as compared to PL intensity.

### 3.2 ML Field to Effective Strain Field Mapping

von Mises stress, or a corresponding effective strain, is considered a trigger for photon emissions from SAO phosphors.^[^ ^16^, ^17^, ^18^, ^19^, ^20^, ^21^, ^23^ ^]^ Correlating the ML intensity of an effective strain is crucial to quantitatively analyze the deformation field via the effects of ML. In this context, the compatibility of ML skin with the DIC method provides a valuable opportunity to calibrate the ML intensity to the effective strain. Mapping of ML intensity to effective strain via uniaxial tension testing can be considered an ideal process owing to the absence of strain concentration sites, simple execution, and straightforward data extraction and processing. It should be noted that both ML intensity and effective strain are scalar quantities.

In general, a sophisticated DIC algorithm is required when using the DIC method to determine the displacement fields and strain field components. In the present study, an open‐source Ncorr 2D DIC MATLAB program was used to obtain the strain field components (*ε*_*xx*_, *ε*_*xy*_, and *ε*_*yy*_) and displacement fields (horizontal field (*u*) and vertical field (*v*)). The DIC algorithm is well documented in ref. [27]. In the present study, several grayscale images from the uniaxial tension test were considered to obtain the displacement field and strain field components, and subsequently differentiate the effective strain field from the strain field components using the relationship specified in Equation (1). To implement the DIC method, we selected a 250 × 28‐pixel ROI from the reference image, as shown in Figure 4a. Furthermore, a 15‐pixel subset radius was set with a subset spacing of 1 pixel and a strain radius of 15 pixels. Ncorr was effectively implemented with an ML skin to determine the displacement fields and strain field components, as shown in Figure 4a and Figure S3 (Supporting Information), respectively. The vertical and horizontal displacement fields and strain field components showed no distortions, highlighting the compatibility of the ML skin with the DIC algorithm. The corresponding effective strain fields of the strain field components are illustrated in Figure 4a. In general, *ε*_e_ is similar to *ε*_*yy*_ because *ε*_*yy*_ is larger than either *ε*_*xx*_ or *ε*_*xy*_; however, the effect of *ε*_*xx*_ and *ε*_*xy*_ on *ε*_e_, and consequently, on triggering of photon emissions, cannot be underestimated

(1) $$\begin{array}{c} \varepsilon_{e}\;=\;\frac{2}{3}\sqrt{\varepsilon_{xx}^{2}\;+\;\varepsilon_{yy}^{2}\;+\;\varepsilon_{zz}^{2}\;-\;\varepsilon_{xx}\varepsilon_{yy}\;-\;\varepsilon_{xx}\varepsilon_{zz}\;-\;\varepsilon_{yy}\varepsilon_{zz}\;+\;3\varepsilon_{xy}^{2}} \end{array}$$

In the above equation, *ε*_*zz*_ was calculated indirectly using Hook's law for plane stress conditions: $\varepsilon_{zz}\;=\;\frac{-\nu }{\left(1\;-\;\nu \right)}\left(\varepsilon_{xx}\;+\;\varepsilon_{yy}\right)$.

After calculating the effective strain field for each considered image, the ML intensity field for each deformed image was determined by subtracting the background photoluminescence using the reference image. It should be noted that the region selected for the subtraction of the deformed image was determined based on *u* and *v* information, which enabled tracking of the reference image ROI in the deformed image. Figure 4a shows the ML intensity field of the deformed image. Both the effective strain field and ML intensity field exhibit variations in the local distribution despite uniaxial loading and these fields are similar at the local level, demonstrating that the effective strain is a trigger for ML photons.

Before correlating the effective strain and ML intensity, the effective strain and ML intensity matrix dimensions were equalized using the bicubic interpolation method.^[^ ^28^ ^]^ Instead of pixel‐to‐pixel mapping between the effective strain and ML intensity, the average value obtained from a 30 × 30‐pixel region was mapped for five subset windows, as illustrated in Figure 4b. Finally, a calibration curve was obtained from the linear regression fit of the scatter plot between the ML and the *ε*_e_ values obtained from each image, as shown in Figure 4c. The curve shows that the ML intensity increases linearly with an increase in the effective strain. Linearity is a crucial property in deformation measurements. However, most conventional strain sensors exhibit linearity only up to the elastic limit, followed by nonlinearity, which renders sensor calibration difficult and reduces the reliability of large deformation measurements.^[^ ^29^, ^30^ ^]^ The linearity in the ML response indicates that strain transfers from the AA7075‐T6 surface to the matrix and from the matrix to the SAO particle are linearly proportional despite the plastic flow in the AA7075‐T6 and in the matrix (Figure S4, Supporting Information). In addition, the linearity in the ML is expected to originate from the elastic deformation of the SAO particles because the stress accumulated in the epoxy matrix is likely to be insufficient to initiate plastic deformation in the SAO particles, which are highly rigid compared with the matrix (*E*_SAO_/*E*_matrix_ ≈ 67, where *E* is the Young's modulus). Because the SAO particles are deformed within the elastic range and strain transfer from the matrix to SAO increases proportionally, the equilibrium of trapping (continuous UV excitation) and detrapping (linear deformation) of electrons in the defect sites of SAO is expected to contribute to the linear response of ML throughout the elastic–plastic deformation of the matrix.^[^ ^8^, ^24^ ^]^

In this framework, calibration between ML intensity and effective strain through a simple tension test can be used to quantify the ML fields in complex geometrical structures and strain amplifying regions such as cracks and holes. Section 3.3 describes the usefulness of a calibration curve for quantifying the evolution of the ML field in the vicinity of the strain amplifying crack tip. In addition, the usefulness of the DIC method for direct visualization of the evolution of the displacement fields, strain field components, and effective strain field in the vicinity of the strain amplifying crack tip was evaluated, as presented in Section 3.3.

### 3.3 Visualizing Crack‐Tip Effective Strain Field

**Figure 5:**
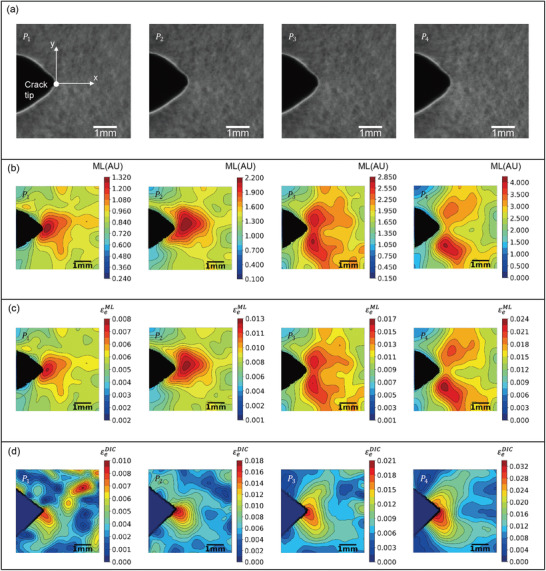
Visualization of the effective strain field at the crack‐tip vicinity in terms of ML effects, quantified ML effects, and DIC measured effective strain. a) Sequential images of a CTS specimen at a constant loading rate of 0.05 mm s^−1^ recorded using a high‐speed camera with a frame rate of 125 fps. b) Sequence of ML images corresponding to each image in (a) illustrating the isointensity contour plots at the crack‐tip vicinity after removing background photoluminescence. c) Sequence of the effective strain contour plots obtained from the pixel‐to‐pixel transference of the ML intensity of (b) using the calibration curve. d) Sequence of the effective strain contour plots obtained from the DIC method using the images in (a).

Many studies have carried out visualization of the evolution of the crack‐tip ML field, and many researchers used UV preirradiation to study the ML field at the crack‐tip vicinity in a dark environment.^[^ ^16^, ^17^, ^18^, ^19^, ^20^, ^21^, ^23^ ^]^ In general, the ML field in the crack‐tip vicinity is brighter than that at the far field, with the brightest point corresponding to the location of the crack tip. The ML emission from the crack‐tip vicinity increases with the increase in the crack‐tip deformation, and at a certain deformation level, the emission can be considerably higher than the light sensitivity threshold of the human eye, as shown in Figure 1c. Notably, the advantage of using UV preirradiation to directly visualize the crack‐tip ML field in a dark ambient environment is lost under UV irradiation, as illustrated in **Figure** **5**a. One reason for this phenomenon is that extremely high background photoluminescence under UV irradiation can nullify the impact of ML emissions during direct visualization of a crack‐tip ML field. The ability to visualize the crack‐tip ML field, however, can be restored by removing the background photoluminescence, as carried out in the calibration step. To visualize the evolution of the ML field in the crack‐tip vicinity, we selected four grayscale images from the fracture test of the CTS specimen at different external loads, as shown in Figure 5a. The load–displacement curve of the fracture test is shown in Figure S5a (Supporting Information), where *P*_1_, *P*_2_, *P*_3_, and *P*_4_ denote the positions of the selected gray images. As illustrated in Figure 5b, visualization of the ML field with the background photoluminescence subtracted can efficiently clarify its evolution in the vicinity of the crack tip as a response to crack‐tip deformation. Furthermore, both the magnitude of the intensity and the size of the intensity field increase with the increase in crack‐tip deformation in response to an externally applied load, as illustrated in Figure 5b. The intensity field also indicates that a transformation occurs in the deformation field near the crack tip with an increase in the external load. At a lower load (*P*_1_), an oval‐shaped deformation field occurs in the vicinity of the crack tip, whereas when the external load increases to that at *P*_3_, a butterfly shaped deformation field first appears at the crack tip. Finally, two distinctive deformation fields advance from the crack tip at an angle of 60° from the crack axis (*x*‐axis). In fact, transformation from the effect of ML is directly related to the deformation mechanism of AA7075‐T6. At a lower load, a small plastic zone develops in the crack owing to the evolution of scattered dislocation at the crack tip, which is surrounded by an elastic strain field. As the external load increases, shear and slip bands start to develop owing to strain localization and these increase at certain angles to the original crack axis until the limit of the fracture toughness is reached. The presence of localized shear bands was verified from the optical microscope image shown in Figure S5b (Supporting Information). Obviously, the ML skin serves as a display system and can help clarify the complex deformation advancement in the structure.

ML effects can provide sufficient qualitative information regarding the origin and evolution of a crack‐tip deformation field and can help track the crack‐tip position and identify the loading modes. However, the mechanical parameters that determine the current status of the structural integrity cannot be quantified by simply using ML effects. The ML field must be quantified to ensure that the ML method can be used to both qualitatively and quantitatively assess the crack‐tip deformation field. In this context, the proposed calibration relationship can be used to quantify the ML field in terms of the effective strain field by converting each pixel intensity value into its corresponding effective strain, as illustrated in Figure 5c. The transferred effective strain field ($\varepsilon_{e}^{\mathrm{ML}})$ can quantify the ML field with a high degree of resemblance to the ML field, which can be attributed to the linearity in their correlation. The quantified ML field can be used to extract the fracture parameters, as described in Section 3.4. Qiu et al. recently reported dynamic visualization of strain distribution and fatigue crack propagation using an organic mechanoresponsive aggregation‐induced emission (AIE) luminogen but they struggled to quantify the ML effects.^[^ ^19^ ^]^ In this regard, the present study could be useful in converting ML effects of several organic/inorganic ML materials.

Calibration facilitates ML quantification in the crack‐tip deformation field. The adopted process, however, was an indirect method because uniaxial tension test data were used to quantify the crack‐tip deformation field. However, quantification was accomplished in real time owing to a reduction in the number of image processing steps and the absence of a complex algorithm. Nonetheless, direct correlation of the ML intensity and effective strain at the pixel level in a complex deformation field such as at the crack tip of an elastic–plastic material can enable reliable evaluation. To demonstrate the feasibility of this process, we used the DIC software and parameters used in the tension test to evaluate the effectiveness of the DIC method in terms of measuring the displacement fields and strain field components at the crack tip using the images shown in Figure 5a. As expected, the DIC‐measured displacement fields and strain field components were smooth with no distortions at the crack‐tip vicinity, as illustrated in Figure S6 (Supporting Information), which demonstrates the importance of using ML skin with the DIC method even in the vicinity of the crack tip. Furthermore, the effective strain field ($\varepsilon_{e}^{\mathrm{DIC}})$ was directly obtained from the DIC‐measured strain field components and shown to be smooth and free of distortions in the crack‐tip vicinity, as illustrated in Figure 5d, although certain differences were observed compared with $\varepsilon_{e}^{\mathrm{ML}}$ (Figure 5c). Specifically, the maximum effective strain point is ahead of the physical crack tip in all instances of $\varepsilon_{e}^{\mathrm{ML}}$, and the point moved in response to the applied load. The maximum point indicates the location of the virtual crack tip, as suggested by Irwin in 1957.^[^ ^31^ ^]^ However, for $\varepsilon_{e}^{\mathrm{DIC}}$, the maximum effective strain point lies at the physical crack tip. Furthermore, $\varepsilon_{e}^{\mathrm{ML}}$ exhibited high sensitivity by displaying the deformation field with high resolution, even at a lower load level (*P*_1_), unlike $\varepsilon_{e}^{\mathrm{DIC}}$. Figure 5c shows the evolution of the shear band in a clearer image than that shown in Figure 5d. Nonetheless, the DIC method can yield important mechanical parameters such as the displacement fields and strain components at the pixel level, which cannot be obtained from the ML effects. However, the effectiveness of the DIC method can be enhanced by using an algorithm that is more suitable for ML skin and by employing a high‐resolution imaging system. These should be examined in future studies. Reliable and novel structural health monitoring can be achieved by analyzing the similarity of the results obtained using the two methods.

### 3.4 Determination of the *J*‐Integral

**Figure 6:**
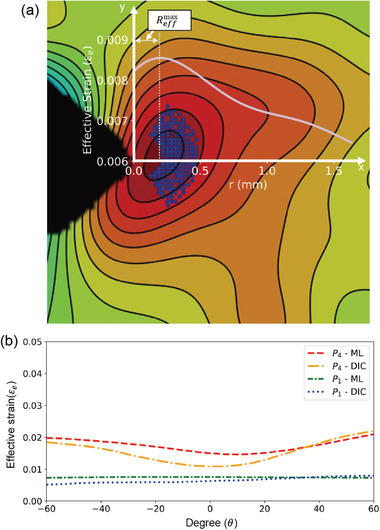
a) Illustration of the zone (white dots) within the HRR field from where more than 1000 effective strain values were selected along with their polar coordinate values to determine the *J*‐integral. The effective strain profile as a function of the distance from the crack tip is also shown in the figure, which indicates that the maximum value of the effective strain is located slightly ahead of the physical crack tip. b) Angular distribution of the average value of the effective strain.

The fracture parameter, known as the *J*‐integral, was determined from both $\varepsilon_{e}^{\mathrm{ML}}$ (Figure 5c) and $\varepsilon_{e}^{\mathrm{DIC}}$ (Figure 5d) using the HRR near‐tip singularity solution.^[^ ^32^, ^33^, ^34^, ^35^ ^]^ The HRR near‐tip singularity solution can determine the *J*‐integral from the effective strain field or from the separate strain field components, whereas the line integral method would require strain components as well as the effective strain. Because the ML field can only be mapped to an effective strain field, the HRR near‐tip singularity solution is a more suitable method to determine the *J*‐integral from the ML field. The effective strain HRR near‐tip singularity solution considered in this work is given by Equation (S4) in Appendix S1 (Supporting Information). Autonomous software built in Python was used to calculate the *J*‐integral from the given effective strain field, crack‐tip location, region of data selection, and material parameters listed in Appendix S1 (Table S1, Supporting Information). The region of data selection was specified within $R_{\mathrm{eff}}^{\max}$ ≤ ($R_{\mathrm{eff}}^{\max}$ + 0.25) and *θ*  ≤   ± 60°, as illustrated in **Figure** **6**a, where $R_{\mathrm{eff}}^{\max}$ is the distance between the maximum effective strain point and the physical crack‐tip position. This aspect of data selection was applied to all instances of $\varepsilon_{e}^{\mathrm{ML}}$. It should be noted that the corresponding coordinates of the data points used for $\varepsilon_{e}^{\mathrm{ML}}$ were obtained and applied to $\varepsilon_{e}^{\mathrm{DIC}}$. Otherwise, $R_{\mathrm{eff}}^{\max}$ would be almost equal to zero in $\varepsilon_{e}^{\mathrm{DIC}}$, which could have resulted in a different data selection region compared with $\varepsilon_{e}^{\mathrm{ML}}$. Because the *J*‐integrals obtained from the two methods were compared, it is necessary to constrain the impact of the differences in the polar coordinates on the *J*‐integral. Furthermore, we ensured that the selected data points were within the plastic and elastic boundaries by calculating the theoretical plastic zone size through the mode I stress intensity factor (SIF) in Irwin's model.^[^ ^31^ ^]^ In addition, the *J*‐integral was obtained via the FEM method using Abaqus 2017 and the values are listed in Table 1 as *J*_FEM_. Details of the FEM method are presented in Appendix S2 (Supporting Information).

The experimentally obtained *J*‐integrals, *J*_ML_ and *J*_DIC_, showed good agreement at lower external loads (*P*_1_); however, once the external load reached the fracture limit, the values deviated by ≈7 kJ m^−2^. This clearly shows that the methods agree at lower levels of crack‐tip deformation but significantly deviate at the point of severe crack‐tip deformation. The agreement at a lower external load and deviation at a higher external load can be attributed to the similarities and dissimilarities in the distribution of the effective strain field. To elucidate this, we determined the average value of the effective strain within the region considered for the *J*‐integral calculation for a given angle and plotted this against the angular coordinate, as shown in Figure 6b. The figure indicates that the average values of the effective strain for both $\varepsilon_{e}^{\mathrm{ML}}$ and $\varepsilon_{e}^{\mathrm{DIC}}$ almost coincide at *P*_1_, but significantly differ at *P*_4_, resulting in mismatched values for the *J*‐integral. The good agreement between *J*_FEM_ and *J*_ML_ (as given in Table 1) throughout the load range confirms that the transferred effective strain field is more accurate and reliable than the DIC‐measured effective strain field for large crack‐tip deformations.

Theoretically, the effective strain fields obtained at the crack‐tip vicinity from the ML and DIC methods should have been identical because the same image was processed for both methods. However, regarding the field morphology, the ML‐based effective strain field illustrated more detailed information, such as virtual crack‐tip position and strain localization pattern. Moreover, the evolution of the effective strain field was vividly visualized even at low‐scale deformation in the ML method. Furthermore, the *J*‐integral from the ML method agrees well with the *J*‐integral from the FEM method. Comparatively poor performance of DIC in the vicinity of the crack tip has been a persistent problem for the DIC method and can be attributed to complexities in the mechanical deformation in the crack‐tip vicinity, which stem from stress triaxiality, strain localization, and a discontinuous displacement field.^[^ ^36^, ^37^ ^]^

Another reason could be that the DIC software used in this work may not be appropriate for ML skin. Customizing the current DIC software or selecting more appropriate DIC software should be examined in future studies. The superior performance of the ML method is related to the ML phenomenon, where each ML particle acts as a strain‐sensing probe to depict detailed information on the deformation field through photon emission. Furthermore, fewer image processing steps in ML method could have also played an important role in retaining deformation information as compared to complex image processing steps in DIC method to provide the location of virtual crack‐tip position and shear band pattern. Nonetheless, reliable and novel structural health monitoring can be achieved by analyzing the similarity of the results obtained using the two methods.

We demonstrated a potential calibration method that converts mechanically induced photons from ML skin into an effective strain using the DIC method by leveraging the inherent intensity pattern of ML skin under UV exposure. The calibrated curve can be used to quantify the ML fields in both simple and complex geometrical structural components, as exemplified by the singularity‐dominated strain field of CTS specimens. Moreover, the essential mechanical parameters, such as the *J*‐integral, that determine the failure state of the structure can be determined using the quantified ML field and DIC‐measured deformation field, with the required data for both methods available in the same photographic image. Indeed, combining two different full‐field deformation measurements has become a hot research topic as this approach improves reliability. For instance, research has been conducted to combine the DIC and IRT methods.^[^ ^38^ ^]^ The requirement of two imaging systems, however, contrasts with the simple coupling of DIC and ML, and can introduce complexities in data acquisition and image processing, thereby increasing the financial burden. In this context, the compatibility of the ML skin with the DIC method offers an opportunity to develop a novel strain sensing method in which the strain sensing performances of both methods can increase the reliability of deformation measurement and help clarify the local and global deformation mechanisms without any extra computational and financial cost. In addition, because both methods use pixel level information of the same photograph, it is expected that correlating the ML intensity with the DIC‐measured displacement fields, strain field components, and effective strain field at the pixel level can elucidate the ambiguous trap‐controlled mechanism of the ML material, which is important for the development of multifunctional advanced ML materials.^[^ ^8^ ^]^

## 4 Conclusion

In an attempt to resolve the quantification problems, we discovered that ML skin fabricated from SAO:Eu,Dy ML microparticles and plasticized epoxy matrix can be subjected to UV exposure and used to monitor ML phenomenon as well as measure the displacement fields, strain field components, and effective strain field using the DIC method. Therefore, we leveraged the compatibility of ML skin with the DIC algorithm to quantify the ML effects that were achieved using pixel level effective strain and the ML intensity information stored in the same photographic image. The results show a linear relationship between effective strain and ML intensity despite the plastic flow in ML skin. The usefulness of the calibrated curve between the effective strain and ML intensity was demonstrated by quantifying the singularity‐dominated ML field at the crack‐tip vicinity of an aluminum alloy (AA7075‐T6, which is commonly used in the aerospace and automobile industries). Further, the possibility of determining the structural integrity parameters such as the *J*‐integral from the quantified ML field was demonstrated using the HRR near‐tip singularity solution and was verified using the FEM method. In addition, the possibility of extending the DIC method to directly measure the singularity‐dominated effective strain field and determine the structural integrity parameters was crucial in validating the measurement using the ML method. The ML skin used in this research can also work within the framework of conventional ML technology where UV is turned off after preirradiation. The compatibility of ML skin with the DIC algorithm not only enables the quantification of the ML effects of several organic/inorganic ML materials, but may also be useful in elucidating the fundamentals of trap‐controlled mechanism, which is important for the development of multifunctional advanced ML materials. The current work is limited to static crack, and further studies on the elastic‐plastic field evolution accompanied in advancing cracks during fatigue test are under investigation.

## Conflict of Interest

The authors declare no conflict of interest.

## Supporting information

Supporting Information

## Data Availability

The data that support the findings of this study are available from the corresponding author upon reasonable request.
