# Supplementary material for: Digital Image Correlation Compatible Mechanoluminescent Skin for Structural Health Monitoring
Source: Adv Sci (Weinh). 2022 Feb 13;9(11):2105889. doi: 10.1002/advs.202105889 (PMC9008412; doi:10.1002/advs.202105889)
Supplement: Supplementary file 1 — Supporting Information [file ADVS-9-2105889-s001.pdf]

ADVANCED SCIENCE
Open Access

Supporting Information

for *Adv. Sci.*, DOI 10.1002/advs.202105889

Digital Image Correlation Compatible Mechanoluminescent Skin for Structural Health Monitoring

Ho Geun Shin, Suman Timilsina, Kee-Sun Sohn and Ji Sik Kim**

ADVANCED SCIENCE
Open Access

Supporting Information

for *Adv. Sci.*, DOI: 10.1002/advs.202105889

Digital Image Correlation Compatible Mechanoluminescent Skin for Structural Health Monitoring

Ho Geun Shin, Suman Timilsina, Kee-Sun Sohn and Ji Sik Kim**

Supporting Information

Digital Image Correlation Compatible Mechanoluminescent Skin for Structural Health Monitoring

Ho Geun Shin, Suman Timilsina, Kee-Sun Sohn and Ji Sik Kim**

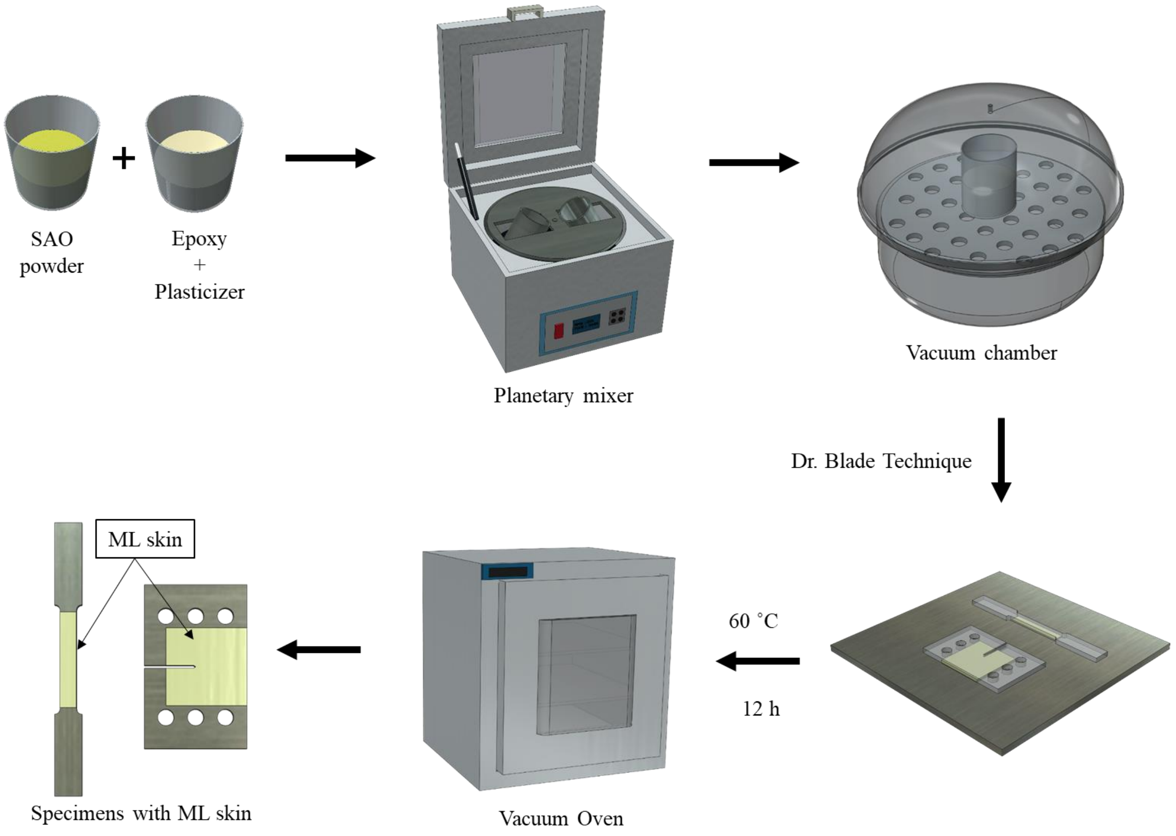

Figure S1. Illustration of ML skin fabrication on the surfaces of the tension and CTS specimens based on the doctor blade technique using a liquid composite comprising SAO powder, epoxy, and plasticizer. Scotch tape with a thickness of 60 µm was used to separate the region of interest in the CTS and tension specimens before spraying with the liquid composite.

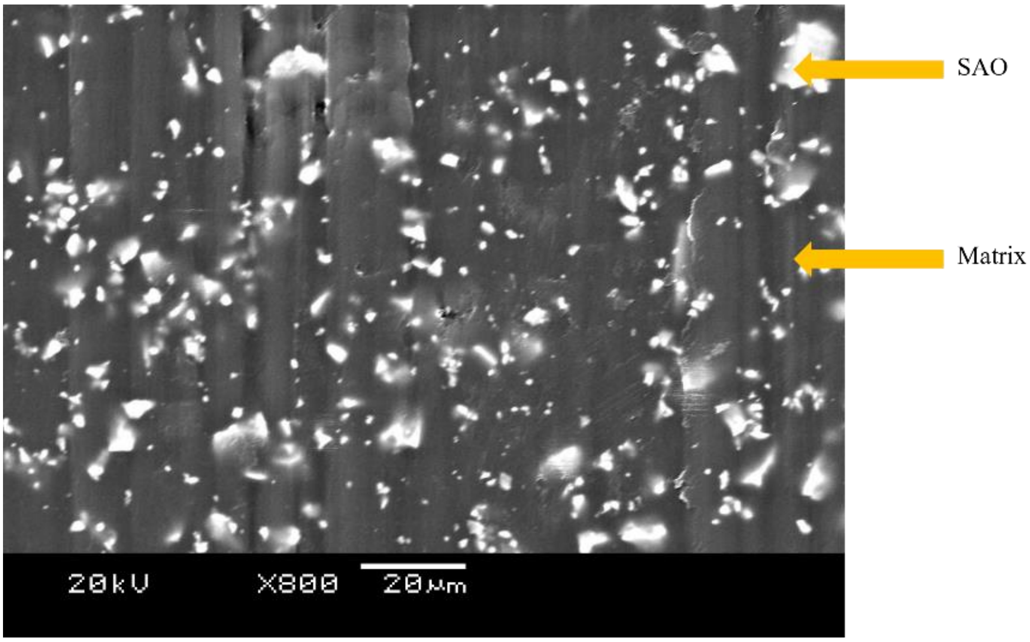

Figure S2. SEM image of the ML skin showing the SAO particle distribution in the epoxy matrix; it depicts the variations in the particle size distribution as well as variations in the dispersion at the microlevel.

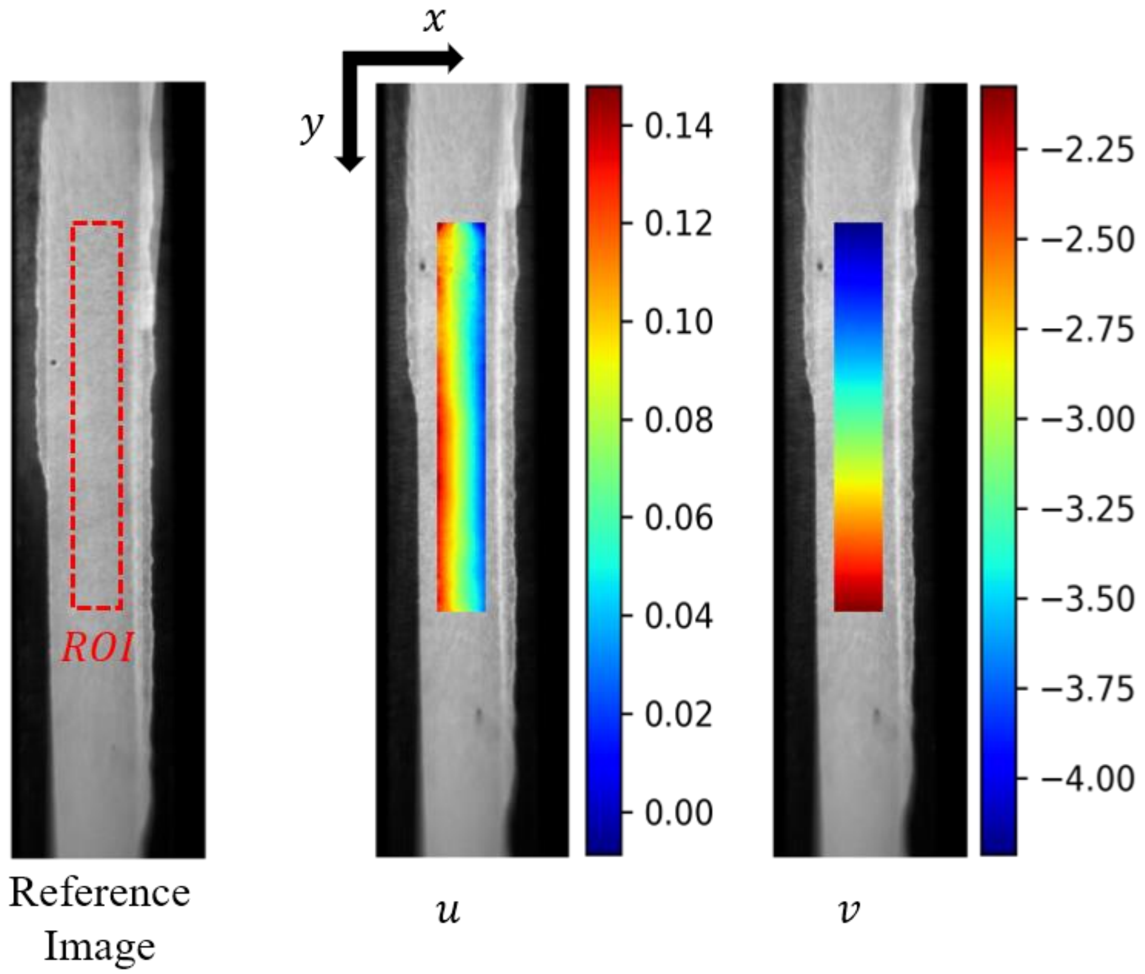

Figure S3. Illustration of the horizontal (u) and vertical displacement (v) fields in the gauge section of the tension specimen. The region of interest (ROI) is shown in the reference image. x and y represent the Cartesian coordinate system adopted in the Ncorr DIC software.

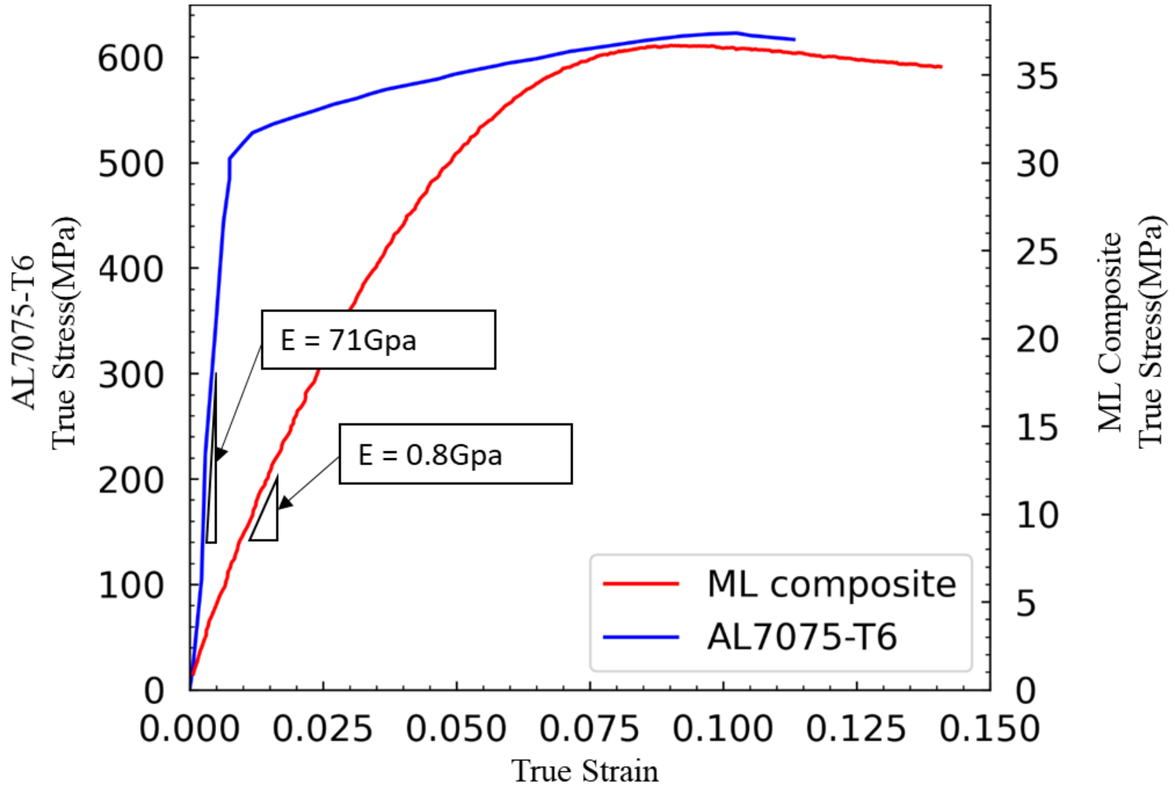

Figure S4. Caparison of the flow curves of the ML composite and Al7075-T6. Both flow curves exhibit plastic deformation.

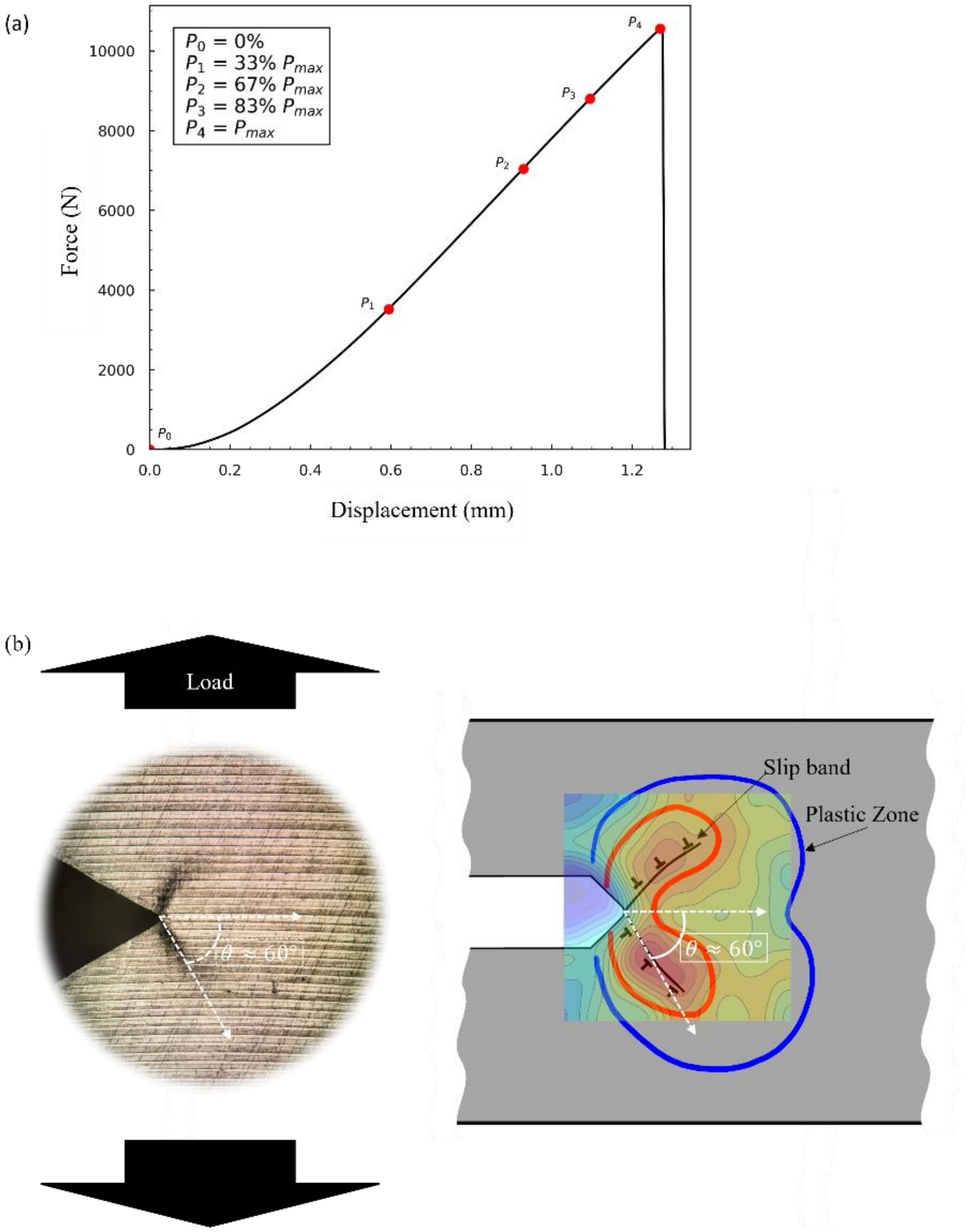

Figure S5. (a) Load–displacement curve of the CTS specimen loaded with a constant crosshead speed of 0.05 mm/s. (b) The optical microscope image illustrates the well-developed slip band at the crack tip vicinity of AA7075-T6. The shape and orientation of the slip band show good agreement with the ML patterns.

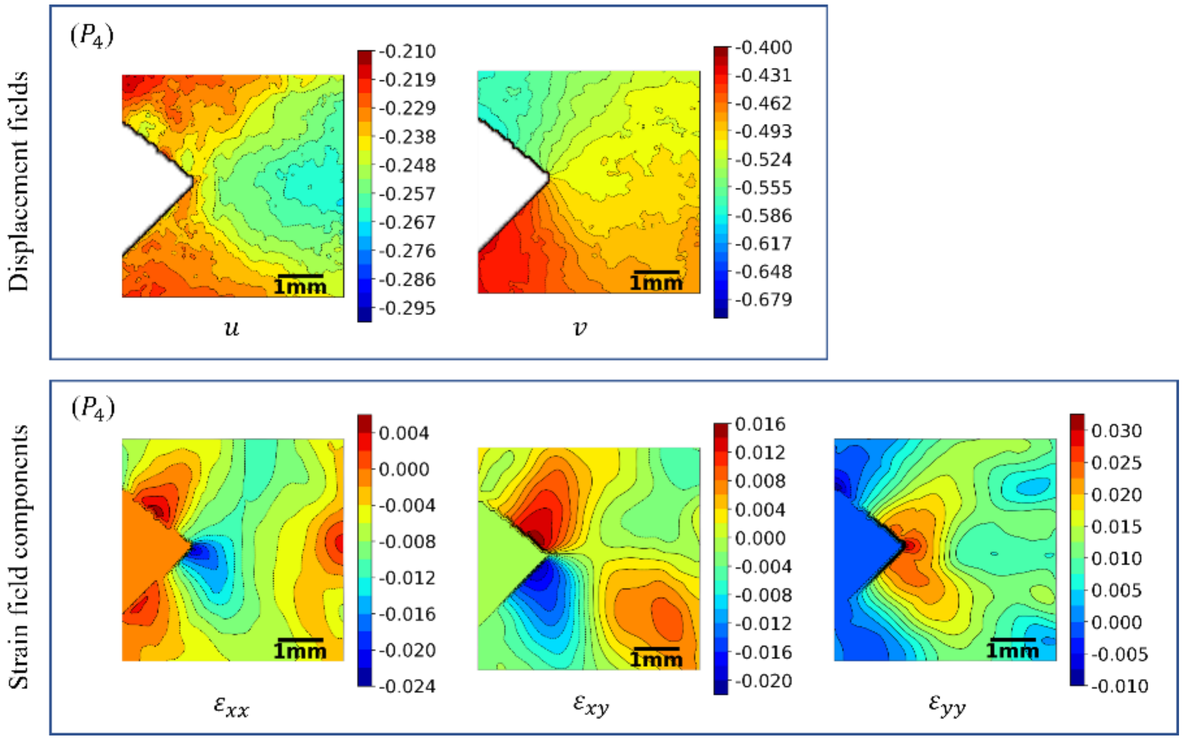

Figure S6. Illustration of the horizontal (u) and vertical displacement (v) fields along with the strain field components (ε_{xx}, ε_{xy}, and ε_{yy}) at the crack tip vicinity of P_4.

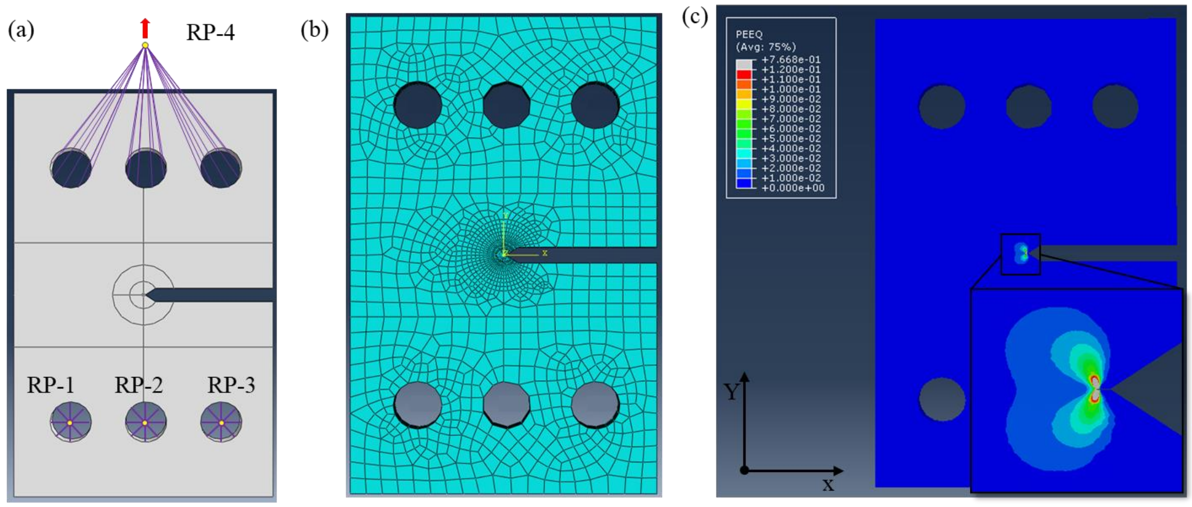

Figure S7. (a) Model of the CTS specimen with the applied boundary conditions. The reference points RP-1, RP-2, and RP-3 were coupled with the surface of the holes and constrained in all directions except for rotation in the out-of-plane direction. The reference point RP-4, which was coupled with the surface of the holes as illustrated in Figure S7(a), was constrained in all directions except for rotation in the out-of-plane direction and movement in the y-axis. (b) Mesh distribution. (c) Simulation of the effective strain distribution.

Appendix 1

Crack tip solution to determine the J-integral

Mechanical parameters independent of the component geometry, crack size, and load configuration are essential for monitoring the structural integrity of components. To satisfy such a requirement, it is essential to assess the crack tip field using crack tip field solutions in which some mechanical parameters dominate the deformation field. In 1952, Williams proposed an asymptotic crack tip solution based on linear elastic fracture mechanics in which the stress field distribution at the crack tip vicinity can be simply expressed in terms of the stress intensity factor (SIF) and the functions that depend on the polar coordinates.[35]
Because William's crack tip solutions work best in the elastic stress and strain fields, Hutchinson proposed asymptotic crack tip solutions that describe the deformation field within the plastic zone for a nonlinear Ramber–Osgood material.[32] Hutchison, Rice, and Rosengren subsequently showed that a J-integral such as the SIF in an elastic field characterizes the plastic field at the crack tip in a power-law hardening material.[33] Hutchison, Rice, and Rosengren proposed asymptotic crack tip solutions that work the best at certain regions within the plastic zone, and this region is known as the HRR field.
The HRR singularity field solutions for stress, strain, and displacement are given by Equations (2a), (2b), and (2c), respectively. [33]

$$\sigma_{ij} = \sigma_0 \left(\frac{J}{\alpha\sigma_0\varepsilon_0 I_n r}\right)^{\frac{1}{n+1}} \bar{\sigma}_{ij}(\theta, n) \tag{2a}$$

$$\varepsilon_{ij} = \alpha\varepsilon_0 \left(\frac{J}{\alpha\sigma_0\varepsilon_0 I_n r}\right)^{\frac{n}{n+1}} \bar{\varepsilon}_{ij}(\theta, n) \tag{2b}$$

$$u_i - \hat{u}_j = \alpha r \sigma_0 \left(\frac{J}{\alpha\sigma_0\varepsilon_0 I_n r}\right)^{\frac{n}{n+1}} \bar{u}_{ij}(\theta, n) \tag{2c}$$

In the above equations, J denotes the J-integral, I_n is a dimensionless parameter, r and θ are the polar coordinates ahead of the crack tip, $\bar{\sigma}_{ij}(\theta, n)$, $\bar{\varepsilon}_{ij}(\theta, n)$, and $\bar{u}_{ij}(\theta, n)$ are dimensionless functions, α is the material property, and σ_o and ε_o are the yield stress and yield strain, respectively.

The effective strain fields illustrated in Figure 4(c) and (d) are very important for determining the SIF and J-integral using the elastic effective strain field and the HHR effective strain field, respectively. In this work, the HHR field was considered to determine the J-integral rather than using the elastic field to determine the SIF because qualitative and quantitative assessment of the plastic zone is considered to be a challenging task in the field of fracture

mechanics.

The HRR crack tip solution that defines the HHR effective strain field can be derived from the strain components as expressed in Equations (3a), (3b), and (3c).

$$\varepsilon_{rr} = \alpha\varepsilon_0 \left(\frac{J}{\alpha\sigma_0\varepsilon_0 I_n r}\right)^{\frac{n}{n+1}} \bar{\varepsilon}_{rr}(\theta, n) \quad (3a)$$

$$\varepsilon_{\theta\theta} = \alpha\varepsilon_0 \left(\frac{J}{\alpha\sigma_0\varepsilon_0 I_n r}\right)^{\frac{n}{n+1}} \bar{\varepsilon}_{\theta\theta}(\theta, n) \quad (3b)$$

$$\varepsilon_{r\theta} = \alpha\varepsilon_0 \left(\frac{J}{\alpha\sigma_0\varepsilon_0 I_n r}\right)^{\frac{n}{n+1}} \bar{\varepsilon}_{r\theta}(\theta, n) \quad (3c)$$

Combining Equations (2) and (3), the HRR effective strain field for 2-D in a polar coordinate can be determined as given in Equation (4).

$$\varepsilon_{\mathrm{e}} = \alpha \frac{\sigma_0}{E} \left(\frac{J}{\alpha\sigma_0\varepsilon_0 I_n}\right)^{\frac{n}{n+1}} r^{-\frac{n}{n+1}} \bar{\varepsilon}_e \quad (4)$$

In Equation (4), $\bar{\varepsilon}_e = \frac{2}{3}\sqrt{(\bar{\varepsilon}_r^2 + \bar{\varepsilon}_\theta^2 - \bar{\varepsilon}_r\bar{\varepsilon}_\theta + 3\bar{\varepsilon}_{r\theta}^2)}$ is a dimensionless effective strain function that depends on θ and n. $\bar{\varepsilon}_e$ can be obtained from the algorithm developed by Jaroslaw et al. [34]

Table S1. Material properties of AA7075-T6

$\boldsymbol{\sigma_o}$ [MPa]	E [GPa]	α	n	In
535	71	0.585	17	2.78

Appendix 2

FEM method to determine J-integral

The experimentally obtained J-integrals (J_{DIC} and J_{ML}) were compared with the FEM J-integral obtained using Abaqus 2017, where the computation is based on a domain integral method. A 3-D CTS specimen was modeled with the mechanical properties of AA7075-T6. True stress and true strain uniaxial tension tests were also conducted using the model to

ensure elastic–plastic fracture. The structure was modeled considering 20-node quadratic brick finite element (C3D20) types, and a value of 0.25 was used for the mid-side node parameter to ensure a singular stress field at the crack tip. The model, mesh, boundary conditions, and effective strain field distribution are illustrated in **Figure S7**.
